# An Update on Acute Appendicitis in Lebanon: Insights From a Single-Center Retrospective Study

**DOI:** 10.7759/cureus.38792

**Published:** 2023-05-09

**Authors:** Nagham Bazzi, Samer Dbouk, Ahmad Rached, Sadek Jaber, Hala Bazzi, Manal Jrad, Mariam Bazzi

**Affiliations:** 1 Medicine, Lebanese University, Beirut, LBN; 2 General Surgery, Al Zahraa Hospital University Medical Center, Beirut, LBN; 3 Internal Medicine and Dermatology, Saint Charles Hospital, Beirut, LBN; 4 Orthopedics, Lebanese University, Beirut, LBN; 5 Faculty of Science, Lebanese University, Beirut, LBN; 6 Radiology, American University of Beirut, Beirut, LBN; 7 Faculty of Public Health, Saint Joseph University of Beirut, Beirut, LBN

**Keywords:** complicated appendicitis, simple appendicitis, acute appendicitis, lebanon, post-colonoscopy appendicitis

## Abstract

Background

Acute appendicitis is the most common surgical emergency worldwide with scarce reports about its prevalence in the Middle East. To date, no epidemiological article has described the incidence of appendicitis in Lebanon. Our primary objective was to estimate the rate of appendicitis in a single center in Lebanon. Our secondary objectives included identifying differences between simple and complicated appendicitis regarding demographics, pre and postoperative characteristics, and symptoms and signs of appendicitis.

Methodology

A retrospective study was conducted at a single central university hospital in Lebanon. Patients with a clear diagnosis of acute appendicitis were included. Pregnant women, lactating women, patients with organ dysfunction, and patients younger than 18 years old or older than 80 years old were excluded. We reviewed and collected the data of patients who presented to the hospital between November 2018 and November 2019 and November 2020 and November 2021.

Results

A total of 95 patients were included in our study, with 35 women and 60 men. The mean body mass index of patients with simple appendicitis was 19.14 ± 9.66 kg/m^2^ compared to 18.97 ± 10.37 kg/m^2^ in patients with complicated appendicitis (p = 0.94). A total of 42.3% of patients who used antibiotics 24 hours after the operation had simple appendicitis, whereas 20.8% had complicated appendicitis (p = 0.004).

Conclusions

Antibiotic usage and the length of hospital stay were correlated with the severity of appendicitis, as reported in the literature. Further randomized studies with a larger number of patients and covering several hospitals in Lebanon are warranted.

## Introduction

Acute appendicitis (AA) is known to be the most common surgical emergency worldwide [1]. A lower incidence of AA is noted in populations belonging to high socioeconomic classes [2]. Moreover, mortality rates are higher with cases of AA affecting age extremes [1,3]. Factors accounting for an increased risk of complications from AA include extremes of age, duration of symptoms before admission, and availability of general surgeons [4].

The diagnosis of AA can be quite challenging. The 2020 update of the World Society of Emergency Surgery Jerusalem guidelines recommends the use of an individualized approach for the diagnosis of AA and for risk stratification based on sex, age, and symptoms [5]. The management of AA consists of a combination of medical and surgical treatments, that is, emergent appendectomy within 24 hours from admission [5], followed by antibiotics for a variable number of days depending on the presence or absence of complications [6]. Laparoscopic appendectomy is superior to an open approach due to fewer complications and shorter hospital stay [5].

A recent population-based study estimated the incidence of appendicitis in the 21st century to be 100, 105, 150, and 160 per 100,000 individuals in North America, Eastern Europe, Western Europe, and Turkey, respectively [7]. The study reported a stable incidence of appendicitis in Western countries compared with a rising incidence in newly industrialized countries such as those of the Middle East. A need for gathering epidemiological data about appendicitis in the Middle East has been previously reported to better structure healthcare delivery in this region of the world [1]. A study published in the British Journal of Surgery in August 2020 found that the number of surgical procedures performed in Europe decreased by 72.3% during the first eight weeks of the pandemic, with a gradual recovery in the following weeks [8]. Additionally, the first case of COVID-19 was reported in December 2019 [9]. Due to the significant impact of COVID-19 in Lebanon, with maximum restrictions in place between December 2019 and October 2020, we made a deliberate effort to avoid conducting our study during this period. Our primary objective was to provide an update on the rate of AA in a single center in Lebanon. Our secondary objectives were to identify differences between simple and complicated appendicitis regarding demographics, pre and postoperative characteristics, and symptoms and signs of appendicitis.

We believe that studies on the epidemiology of appendicitis are much needed as they will pave the way for future studies on the economic burden of appendicitis, related to appendectomy and hospital stay. The usage of fried and fatty meals in traditional Lebanese cuisine is well known for raising the risk of appendicitis. In addition, the identification of factors related to complicated appendicitis is the first step toward the implementation of strategies aimed at reducing the incidence of complications arising from AA.

## Materials and methods

Study design and ethical approval

A retrospective review of patient charts was performed. Patients admitted with a diagnosis of AA in the period between November 2018 and November 2019 (before the COVID-19 pandemic) and between November 2020 and November 2021 (during the pandemic) were included. Patients were divided into two groups, namely, simple appendicitis and complicated appendicitis. All patients were treated at Al Zahraa University Medical Center in Beirut, Lebanon. Ethical approval was obtained from the Institutional Review Board at Al Zahraa Hospital University Medical Center, Beirut, Lebanon (approval number: 1/2022). Informed consent was not required because there was no risk to participants and the study only conducted a retrospective analysis of patient records.

Inclusion and exclusion criteria

Inclusion and exclusion criteria were similar to a previously published study by Bazzi et al. [10]. Lebanese patients with acute appendicitis aged between 18 and 80 years, non-pregnant or lactating women, and patients without significant organ dysfunction or other surgical contraindications were included in the study. Patients not meeting the inclusion criteria were excluded.

## Results

General characteristics

A total of 95 patients were included in this study, with 35 females and 60 males. Patients with simple appendicitis had a mean age of 33.7 ± 13.3 years, whereas those with complicated appendicitis were older with a mean age of 39.1 ± 13.5 years (p = 0.08). The mean body mass index (BMI) of patients with simple appendicitis was 19.1 ± 9.6 kg/m^2^ versus 18.9 ± 10.3 kg/m^2^ for patients with complicated appendicitis (p value= 0.94). Sex, age, and BMI were statistically non-significant with respective p-values greater than 0.05 (0.06, 0.088, and 0.94, respectively).

The mean C-reactive protein (CRP) level was 111.4 ± 34.1 mg/dL among patients presenting with simple appendicitis versus 150.3 ± 66.5 mg/dL among those having complicated appendicitis. This association was statistically significant with a p-value of 0.03 (<0.05) (Table 1).

**Table 1:** General characteristics of patients. WBC: white blood cells; CRP: C-reactive protein; BMI: body mass index

		Appendicitis				P-value
		Simple		Complicated		
Sex	Female	30	42.3%	5	20.8%	0.06
	Male	41	57.7%	19	79.2%	
Smoker	No	36	50.7%	14	58.3%	0.518
	Yes	35	49.3%	10	41.7%	
Alcoholic	No	68	95.8%	24	100%	0.569
	Yes	3	4.2%	0	0%	
Mean age (years) ± SD		33.7		39.17		0.088
Mean BMI		19.14		18.97		0.94
Mean WBC (mm^3^)		8,907.99		9,522.5		0.688
Mean CRP (mg/dL) ± SD		111.43 ± 34.149		150.31		0.033
Mean neutrophil (%) ± SD		66.008		69.488		0.507

Symptoms and preoperative characteristics

Furthermore, 21.1% of simple ruptured AA cases versus 50% of complicated ruptured AA cases had an intraperitoneal culture showing evidence that simple versus complicated appendicitis is significantly correlated with intraperitoneal culture post-rupture with a p-value of 0.007 (<0.05).

Finally, right lower quadrant pain, migration/periumbilical pain, nausea, fever, and anorexia were the most commonly reported signs and symptoms of AA (Table 2).

**Table 2:** Signs and symptoms of patients.

Symptom/Sign	Complicated	Simple	Total
Post-menopausal abnormal uterine bleed	0	1	1
Abdominal discomfort	0	1	1
Endometrial cancer	0	1	1
Right lower quadrant pain	28	23	51
Anorexia	10	15	25
Nausea	8	16	24
Vomiting	7	12	19
Diarrhea	1	0	1
Fever	5	6	11
Rovsing	3	2	5
Obturator sign	1	0	1
Chills	0	1	1
Guarding	0	4	4
Headache	0	1	1
Migration/Periumbilical pain	0	5	5
Dizziness	0	1	1
Chronic fatigue	0	1	1
Itching	0	1	1
Umbilical hernia	0	1	1

Postoperative characteristics

First, among patients who received antibiotics for 24 hours postoperatively, 42.3% had simple AA and 20.8% had complicated AA. Second, among those who received antibiotics for 48 hours postoperatively, 46.5% had simple AA and 37.5% had complicated AA. Third, among those who received antibiotics for three days postoperatively, 8.5% had simple AA and 29.2% had complicated AA. Finally, those who received antibiotics for five to seven days postoperatively had complicated AA only. This association was statistically significant with a p-value of 0.004 (<0.05) (Table 3).

**Table 3:** Diagnosis, symptoms, and preoperative characteristics.

		Appendicitis				P-value
		Simple		complicated		
Mean duration of symptoms (days)		35.27		83.63		0.13
Mean highest temperature (°C)		37.43		37.925		0.051
Mean duration of preoperative antibiotics use (days)		22.51		20.17		0.587
Way of preoperative antibiotic administration for complex appendicitis at home	None	63	88.7%	21	87.5%	0.87
	PO	8	11.3%	3	12.5%	
Did the patient receive an appendectomy?	No	2	2.8%	0	0%	1
	Yes	69	97.2%	24	100%	
Was an intraperitoneal culture in ruptured appendicitis?	No	56	78.9%	12	50%	0.007
	Yes	15	21.1%	12	50%	

Similarly, among those who had a restoration of their gastrointestinal (GI) transit in one day, 42.3% had simple AA and 12.5% had complicated AA. Among those who had bowel movements in two days, 49.3% had simple AA and 70.8% had complicated AA. Additionally, among those who had GI transit in four days, 4.2% had complicated AA with none belonging to the simple appendicitis group. Moreover, the majority (41.7%) of those who had their abdominal abscess drained had complicated appendicitis primarily. These associations were statistically significant with a p-value of less than 0.05.

The mean hospital stay was 2.69 ± 0.919 days in cases of simple AA compared to 3.88 ± 1.84 days in cases of complicated AA. This association was equally statistically significant with a p-value <0.01 (Table 4).

**Table 4:** Postoperative characteristics. SD: standard deviation; GI: gastrointestinal; AB: antibiotics; IV: intravenous; N/A: not applicable

		Appendicitis				P-value
		Simple		complicated		
Mean operating time (minutes) ± SD		71±66.32		24±77.54		0.134
Duration of postoperative antibiotic use for complex appendicitis	24 hours	30	42.3%	5	20.8%	0.004
	48 hours	33	46.5%	9	37.5%	
	3 days	6	8.5%	7	29.2%	
	5 days/7 days	0	0%	3	12.5%	
	No surgery	2	2.8%	0	0%	
Way of postoperative antibiotic administration for complex appendicitis at the hospital	None	6		0		0.119
	IV	64		24		
	Oral PO	1		0		
Average assumption of GI transit (days)	1 day	30	42.3%	3	12.5%	0.01
	2 days	35	49.3%	17	70.8%	
	3 days	3	4.2%	3	12.5%	
	4 days	0	0%	1	4.2%	
	N/A	3	4.2%	0	0%	
Postoperative complications	Intra-abdominal abscess	0	0%	1(4.2%)		0.06
	None	71	100%	22(91.7%)		
	Patient was febrile	0	0%	1 (4.2%)		
Mean hospital stay (days) ± SD		2.69 ± 0.919		3.88 ± 1.849		<0.001
Did the patient receive abscess drainage?	No	65	91.5%	14	58.3%	0
	Yes	6	8.5%	10	41.7%	
Did the patient receive an appendectomy after AB therapy?	No	45	63.4%	11	45.8%	0.131
	Yes	26	36.6%	13	54.2%	

## Discussion

Age and BMI were not significantly different between those with simple and complicated appendicitis. However, the mean CRP level and the likelihood of intraperitoneal culture post-rupture were significantly higher in those with complicated appendicitis. Right lower quadrant pain, migration/periumbilical pain, nausea, fever, and anorexia were the most commonly reported signs and symptoms of appendicitis. The duration of postoperative antibiotic treatment, restoration of gastrointestinal transit, drainage of abdominal abscess, and length of hospital stay were all significantly associated with the severity of appendicitis. These findings were specific to our institution.

Our findings mirror those of other studies suggesting that higher CRP levels are seen in cases of complicated AA rather than in patients with simple AA [11]. Moreover, CRP levels are precise predictors of appendicitis severity, especially when interpreted with the respective white blood cell and neutrophil counts [12,13].

Antibiotics were used more often in patients with simple appendicitis, whereas in those with complicated appendicitis, antibiotics were selected depending on the degree of contamination. According to other studies, antibiotic therapy is a feasible treatment option for cases of simple appendicitis instead of appendectomy [14-16]. Di Saverio et al. reported in their study that antibiotic treatment for simple appendicitis is safe, effective, cost-effective, and can avoid unnecessary surgery and surgical risk [17]. Additionally, the recurrence of disease symptoms was found to be 14% less among these patients and can be safely and effectively treated [17]. Furthermore, a previous study suggested that multiple antibiotics may not be effective in patients with complicated appendicitis [18]. This clearly matches our findings.

Regarding antibiotics consumption post-appendectomy, complicated cases used antibiotics longer than those with simple appendicitis (average of one to seven days vs. one to three days). According to American guidelines, antibiotic use is advised for four to seven days after complicated appendicitis [19]. Postoperative antibiotics are recommended after appendectomy for complex appendicitis to reduce infectious complications [20].

Moreover, in our study, intraperitoneal cultures were taken more in cases of complicated appendicitis. This is very important to prevent uncommon infections that might lead to more severe symptoms, especially with the increase in drug resistance. Bacteria resisting the chosen antibiotics means more infectious complications and longer hospital stays among patients with appendicitis [21,22]. Our study was conducted during the COVID-19 pandemic which was very challenging for the Lebanese healthcare system that was short on medical equipment including antibiotics [23]. This would better explain the increase in the rate of appendicitis cases complicated by infections.

Furthermore, longer hospital stays were seen in cases of complicated appendicitis rather than in cases of simple appendicitis. This is in line with other studies reporting that patients with complicated AA stay six times longer in hospitals when compared to those with simple AA [24]. Similarly, other studies have shown that a longer hospital stay for complicated appendicitis is associated with reduced postoperative morbidities and wound infection and a quicker return to normal activities [25].

In our study, among those who underwent drainage for an appendiceal abscess, the majority had complicated appendicitis. This is consistent with previous studies stating that patients with complicated appendicitis and who underwent appendectomy are more prone to develop postoperative complications such as abscesses [26]. This can be explained by the Lebanese economic crisis that left people unable to purchase medications capable of limiting their infection, ending up with worse disease and abscesses requiring drainage after appendectomy.

One of the main limitations of our study is collecting data from a single center. Thus, our data cannot be extrapolated to the country, even though it is one of the largest hospitals. Additionally, the COVID-19 pandemic and the retrospective nature of the study are also other limitations. A selection bias could be present due to the loss of follow-up with some of our included patients, and missing data in patient files could have predisposed to information bias.

## Conclusions

CRP levels, antibiotic usage, and length of hospital stay were correlated with the severity of appendicitis, which is similar to other studies reported in the literature. Further randomized studies with a larger number of patients and covering many hospitals in Lebanon are warranted.
